# A rare pituitary tumor associated with hyperthyroidism and acromegaly

**DOI:** 10.3389/fendo.2022.1072647

**Published:** 2022-12-23

**Authors:** Li Li, Liheng Meng, Yuping Liu, Rashi Tamrakar, Xi Yang, Xinghuang Liang, Jia Zhou, Jing Xian, Zhenxing Huang, Yingfen Qin

**Affiliations:** ^1^ Department of Endocrinology, The First Affiliated Hospital of Guangxi Medical University, Nanning, Guangxi, China; ^2^ Department of Geriatric Endocrinology, The First Affiliated Hospital of Guangxi Medical University, Nanning, Guangxi, China

**Keywords:** mixed pituitary adenoma, thyroid stimulating hormone, growth hormone, rare disease, immunostaining

## Abstract

**Background:**

Mixed pituitary TSH/GH adenomas are rare adenomas associated with acromegaly and/or thyrotoxicosis, with or without varying degrees of goiter. In this report, we show a case of pituitary adenoma producing both GH and TSH simultaneously.

**Case presentation:**

A 27-year-old man was diagnosed with pituitary adenoma based on various symptoms and clinical findings. For further examination and treatment, he was hospitalized in our institution. It was likely that this subject had pituitary adenoma producing both GH and TSH. In brain magnetic resonance imaging, there was a giant tumor in the sellar region. After the diagnosis of mixed pituitary TSH/GH adenoma, he was treated with octreotide, then underwent tumor resection, and then received hydrocortisone acetate and levothyroxine sodium. After then, GH and IGF-1 levels were suppressed and thyroid function was normalized. Postoperative immunohistochemistry reports showed GH (+) but TSH (-), which may be insensitive to the antibody used to detect TSH or combined with other factors.

**Conclusions:**

The diagnosis of mixed pituitary TSH/GH adenoma must be combined with clinical manifestations, immunohistochemical staining and relevant hormone levels, and genetic testing if necessary for comprehensive judgment. For patients with large adenomas, it is recommended to use somatostatin analogs to restore TH levels and control the excessive secretion of GH levels before surgery.

## Introduction

Thyrotropin-secreting pituitary adenomas (TSHomas) are extremely rare adenomas encountered in clinical practice, accounting for about 0.5%–3% of all pituitary adenomas (1). TSHomas have an insidious onset and slow progression and often present as macroadenomas. TSHomas can be divided into simple and mixed TSHomas. Mixed TSHomas account for 20%–25% of TSHomas, and approximately one in three co-secrete other anterior pituitary hormones, most commonly growth hormone (GH) and/or prolactin (PRL) (2). Mixed TSHomas secreting thyroid-stimulating hormone (TSH) and GH have various clinical manifestations, often presenting with acromegaly and/or thyrotoxicosis and with or without thyroid enlargement. Presently, many studies have reported poor efficacy and prognosis of mixed pituitary TSH/GH adenomas (3, 4). In this case report, we retrospectively analyzed the clinical features of a case of pituitary adenoma secreting both GH and TSH simultaneously. We summarized the existing literature, which helped deepen the understanding of this rare disease to avoid misdiagnoses and missed diagnoses.

## Case reports

A 27-year-old male patient presented in the outpatient department with a history of enlarged hands and feet, a broad nose and huge tongue for more than 9 years, and prominent cheekbones with high arched eyebrows for more than 5 years. In 2011, he (18 years old) gradually developed enlargement of hands and feet, widening of the nose, enlarged tongue, and thickened lips, accompanied by a deepened and husky voice that gradually worsened over the years. There was no history of joint pain, morning stiffness, mobility restriction, nasolabial bulge, rough skin, and other discomforts. In 2014, he started noticing arched eyebrows, prominent temporal and cheekbones, enlarged and protruded mandible, and weakened strength of teeth bite, accompanied by headache, dizziness, tinnitus, sweating, shaky hands, palpitations, a significant decline in memory, generalized weakness, being easily fatigued, and muscle soreness that increased with activities, increased appetite, decreased libido (ejaculation once in every 2 months, has erection), and gradual decrease in vision without any visual field defects. Pituitary MRI showed a sellar mass suggestive of a pituitary tumor. Based on these findings, he was diagnosed with pituitary adenoma and hospitalized in our institution for further examination and treatment. Since the onset of the symptoms, he had difficulty falling asleep and waking up in the morning, the bowel and bladder movements were regular, and he had gained 6 kg of body weight. He also explained he was not tall enough compared with his colleagues in high school but later grew 4 cm tall during his university days. He had a history of asthma for more than 10 years, with occasional asthmatic attacks till high school. The frequency of asthmatic episodes increased in the university; thus, he started using salbutamol sulfate metered dose inhalers (MDIs) during the acute onsets. In 2013, he underwent septoplasty for “deviation of the nasal septum.” On inquiring about personal history, the patient was a smoker, had been smoking for 14 years, had about 20 sticks cigarettes per day, and was allergic to sulfonamides. Family history was not significant.

On admission, his height and body weight were 177.5 cm and 60.0 kg with a BMI of 19.2 kg/m^2^. His blood pressure and heart rate were 153/101 mmHg and 128 bpm, respectively. Hypertrophy of the extremities with changes in facial morphologies (prominent temporal and zygomatic bone, mandibular prognathism, high arched eyebrows, widening and thickening of the nasal wings, hypertrophic lips, and coarse and thickened skin of the hands and feet) was noticed. The pitch of the voice was low and slightly deepened, and the hands were flattened with fine tremors. There were no abnormalities in heart and lung sound and in the abdomen.

**Figure 1:**
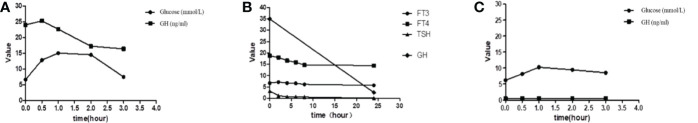
**(A)** Inhibition assay before treatment with glucose. **(B)** Inhibition assay before treatment with somatostatin. **(C)** Postoperative reports of the glucose suppression test. GH, growth hormone; TSH, thyroid-stimulating hormone; FT3, free triiodothyronine; FT4, free thyroxine.

**Figure 2:**
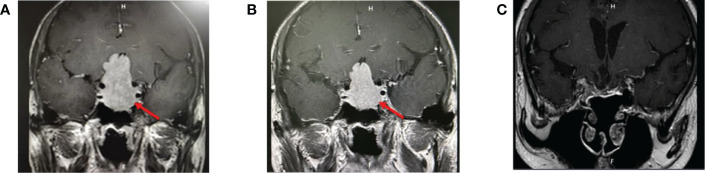
**(A)** Pituitary MRI before treatment: a large mass of equal T1, slightly longer T2 signal foci in the saddle area, clear boundaries, about 2.6 × 2.7 × 4.7 cm in size, protruding upward into the suprasellar cistern, semi-enveloping the internal carotid arteries on both sides, and the optic chiasm pressured upward. **(B)** Pituitary MRI after 6 months of octreotide: similar to that before treatment. **(C)** Pituitary MRI 9 months after surgery.

The data on admission were as follows: GH, 28.036 ng/ml (0.003–0.971); IGF-1, 641.1 ng/ml (60–350); FT3, 10.9 pmol/l (3.28–6.00); FT4, 20.94 nmol/l (7.86–14.41); TSH, 2.29 mIU/ml (0.34–5.65); FSH, 1.75 mIU/ml (0.7–12.4); LH, 2.04 mIU/ml (0.8–7.7); PRL, 14.68 ng/ml (6.58–31.18); progesterone, 0.24 ng/ml (0.1–2.1); testosterone, 0.93 ng/ml (2.27–9.76); estradiol, 18.99 pg/ml (0–84); gonadal hormone-binding globulin (SHBG), 54.4 nmol/l (13.6–76.3); cortisol, 8 am 75.7 nmol/l (185–624), 4 pm 16 nmol/l (107.81–333.64); ACTH, 8 am 30.97 nmol/l (7–65), 4 pm 24.77 nmol/l (3-30); 24 h urinary free cortisol, 136.27 nmol/l (185–624). CRP, AFP, CEA, CA125, CA153, CA199, and PTH were within the normal range. TRAb, TPOAb, and TgAb all showed negative findings. The rest of the laboratory tests are shown in **Table 1** and **Figure 1**. Computerized visual field monitoring showed decreased photosensitivity above the temporal part of both eyes. Ultrasound (US) of the abdomen showed splenomegaly with no other abnormalities. US thyroid scan indicated nodular goiter in the right lobe (TI-RADS classification 3). CT scan of the sinuses and neck detected small cysts under the mucosa of the maxillary sinuses on both sides, deviated nasal septum, incomplete adenoid degeneration, enlarged and calcified tonsils, reactive hyperplasia of lymph nodes in the neck, and a mass in the sellar area, suggestive of a pituitary tumor. Pituitary MRI showed a large mass of equal T1 and a slightly longer T2 signal lesion in the sellar region with a clear boundary, about 2.6 cm × 2.7 cm × 4.7 cm in size, protruding upward into the suprasellar cisterna, semi-encompassing the internal carotid artery on both sides, and the optic chiasm was pressed upward. Imaging diagnosis showed a space-occupying lesion in the sellar region, most likely to be a pituitary tumor (**Figure 2**). A plain CT scan + coronal and sagittal reconstruction of the sellar region was repeated in a tertiary hospital in Beijing that showed an enlarged sella turcica with a depressed floor, a mass (25 mm × 26 mm × 41 mm) with a slightly high-density shadow in the sella turcica with a clear boundary. The lesion protruded upward to the suprasellar cisterna, and the optic chiasm compressed and was pressed upward. The pituitary stalk was not visible, and bilateral cavernous sinuses were compressed, features suspecting a pituitary tumor. DXA measured the bone mineral density of lumbar vertebrae and left hip: the average L1–L4 Z score of the lumbar vertebrae was -3.8, and the Z score of the left hip was -3.1, indicating osteoporosis. Whole exome sequencing of the family (including thyroid hormone receptor β, THR β gene, and mitochondria) showed a negative result.

**Table 1:** Changes in relevant hormone indexes before treatment, after octreotide, and after surgery.

	Before treatment	After 6 months of octreotide	The 3rd day after surgery	The 4th month after surgery	Reference range
GH (ng/mL)	28.036	1.057	0.081	0.24	0.003–0.971
IGF-1 (ng/mL)	641.1	189.70	–	68.99	60–350
TSH (mIU/mL)	2.29	0.28	0.018	0.31	0.34–5.65
FT4 (pmol/L)	20.94	9.19	9.34	10.37	7.86–14.41
FT3 (pmol/L)	10.90	4.01	2.57	3.38	3.28–6.00
FSH (mIU/mL)	1.75	2.52	0.78	1.99	0.7–12.4
LH (mIU/mL)	2.04	3.07	0.71	2.41	0.8–7.7
PRL (ng/mL)	14.68	17.36	<0.5	1.29	6.58–31.18
P (ng/mL)	0.24	0.16	<0.2	0.22	0.1–2.1
T (ng/mL)	0.93	1.79	<0.2	0.64	2.27–9.76
E2 (pg/mL)	18.99	27.45	21.45	51.40	0–84

GH, growth hormone; IGF-1, insulin-like growth factors-1; TSH, thyroid-stimulating hormone; FT3, free triiodothyronine; FT4, free thyroxine; F, cortisol; FSH, follicle-stimulating hormone; LH, luteinizing hormone; PRL, prolactin; P, progesterone; T, testosterone; E2, estradiol.

Before the surgery, he was treated with sustained-release metoprolol succinate to control the heart rate, hydrocortisone acetate replacement therapy, and salmeterol fluticasone MDI to control asthma episodes. Before the use of long-acting somatostatin, the patient’s blood glucose level fluctuated between 9 and 11.2 mmol/l after breakfast and between 10 and 13.5 mmol/l after lunch. Therefore, insulin 3 U was given through subcutaneous injection before breakfast and lunch. After the first dose of long-acting growth hormone, the blood sugar level was maintained in the normal range, so insulin was discontinued and blood sugar was controlled through diet instead. Starting on 16/102020, the patient received a monthly injection of 20 mg of octreotide acetate, but his IGF-1 level did not decrease significantly, so we increased it to 30 mg. The relevant hormone indexes after the treatment are shown in **Table 1**. At the same time, the hypermetabolic symptoms of thyrotoxicosis such as increased appetite, sweating, palpitations, hand tremor, and headache were significantly improved, and the blood pressure and blood glucose level were also stable. In August 2021, the patient underwent endoscopic transnasal tumor resection surgery in a hospital in Beijing. The tumor was resected completely, and postsurgery, he was treated with hydrocortisone acetate and levothyroxine sodium. Postoperative immunohistochemistry reports showed GH (+), PRL (scattered in small amounts +), LH (+), TSH (-), ACTH (-), Ki-67 (even +, <1%), PIT-1 (+), SF-1 (+), and T-PIT (-). Pathological diagnosis of pituitary adenoma was made with immunohistochemistry reports of positive GH, luteinizing hormone (LH), and scattered positive prolactin (PRL). The results of the glucose suppression test on the 7th day postsurgery are shown in **Figure 1C**. After the surgery, the patient was treated with levothyroxine, steroid, and antihypertensive medications. Muscle weaknesses were observed. The power of the left lower limb muscle was 1, and that of the left upper limb was 2. The CT scan of the head and MRI plain and enhanced scan + DWI + FLAIR of the brain were done, which denoted cerebral infarction in the anterior cerebral artery on the right side. The patient was then referred to the local rehabilitation center for rehabilitation therapy following his discharge from the hospital. Postoperative reports of the relevant hormones are shown in **Table 1**.

## Discussion

Mixed pituitary TSH/GH adenoma can secrete TSH and GH simultaneously, which is a very rare mixed pituitary adenoma. As far as we know, 55% of pituitary adenomas have been reported to secrete approximately two to three hormones at the same time, among which 42% produce GH and prolactin, 7% produce GH and TSH, and 6% produce GH, TSH, and prolactin (3, 5). Some studies have reported that the clinical manifestations of mixed pituitary TSH/GH adenomas mainly include hyperthyroidism with or without goiter and the mass effects of pituitary adenomas such as headache, vision loss, visual field defects, and acromegaly (3, 6). Although hyperthyroidism is often the first manifestation of this disease, thyroid autoantibodies are primarily negative and thyroid diseases such as exophthalmos, mucous edema, and diffuse goiter caused by thyroid ophthalmopathy generally do not occur. Therefore, the symptoms of hyperthyroidism in some patients may be milder and are easily ignored. Similarly, due to the absence of self-awareness of the symptoms in the early stage of acromegaly, it often takes many years to recognize the series of typical symptoms and signs of elevated GH. Therefore, clinicians need to be vigilant to prevent misdiagnosis and mistreatment.

Since mixed pituitary TSH/GH adenomas often present with hyperthyroidism as the first clinical manifestation, the patient’s serum-free TH is elevated; however, the TSH level remains unrepressed. Some patients with severe hyperthyroidism symptoms are easily misdiagnosed as Grave’s disease (7). Antithyroid drugs and/or iodine 131 therapy can further promote TSH secretion, resulting in further enlargement of the adenoma. They are usually manifested as macroadenoma on imaging (7, 8). Therefore, if clinicians encounter patients with hyperthyroidism symptoms with elevated free TH but normal TSH levels and negative thyroid antibody, they should ruminate on the syndrome of inappropriate secretion of TSH (SITSH). In addition to abnormal TH transporters, the presence of interfering substances in the serum, and different stages of thyroid disease or drug interference, it is crucial to identify the cause of SITSH. SITSH is mainly caused by TSHomas secreting excessive TSH or syndromes of resistance to thyroid hormone (RTH), causing the pituitary gland to be insensitive to TH. The differential diagnoses of patients with excessive secretion of TSH by RTH and mixed pituitary TSH/GH adenomas, as reported by relevant literature (2, 3), are as follows. ① Family history: RTH patients often have a significant family history, whereas mixed pituitary TSH/GH adenomas are mostly sporadic cases, often without any family history. ② Clinical features: Due to the combined resistance of the surrounding pituitary tissues, the symptoms of hyperthyroidism in RTH patients are usually not obvious and even the clinical manifestations of hypothyroidism may occur. However, the symptoms of hyperthyroidism in patients with mixed pituitary TSH/GH adenomas may be more severe due to excessive secretion of TSH. On examination of the thyroid gland, patients with RTH and TSHomas can have varying degrees of goiter. However, it is essential to note that mixed pituitary TSH/GH adenomas can oversecrete GH, which can further cause goiter. The mechanism may be related to IGF-1, increased cell proliferation, and decreased apoptosis (9, 10). ③ Laboratory reports: The concentration of serum SHBG and glycoprotein hormone α subunit (α-GSU) can be increased in patients with TSHomas. In contrast, the serum SHBG and α-GSU concentrations are usually normal or decreased in patients with RTH. Mutations in the THR β gene can be found in 85% of patients with RTH but are not detected in patients with TSHomas (8). Pituitary MRI in RTH usually does not have positive findings, whereas TSHomas often have positive results. ④ Endocrine test: After exogenous L-T3 (i.e., T3 inhibition test), RTH patients usually show partial inhibition of TSH, whereas patients with TSHomas show no inhibition. In the TRH stimulation test, the reaction of RTH patients was normal or enhanced, whereas TSHoma patients showed no response. In the somatostatin inhibition test, RTH patients may not show any suppression but most TSHoma patients show significant inhibition. In 2015, Ruijin Hospital reviewed and analyzed 61 RTH patients; only five had positive MRI findings. Hence, when clinical suspicion of pituitary TSHomas is not supported by imaging, further TRH stimulation tests should be recommended (11).

In this case, the patient had clinical features of hyperthyroidism and acromegaly, thyroid autoantibodies showed negative findings, pituitary MRI showed positive findings, THRβ gene detection showed negative findings, and GH, IGF-1, TH, and TSH levels were significantly reduced after multiple uses of long-acting somatostatin analogs prior to surgery. Hence, the qualitative localization diagnosis of mixed pituitary TSH/GH adenoma was considered. However, it is necessary to be vigilant that pituitary TSHomas combined with RTH can occur in a few cases. Although postoperative immunohistochemistry showed positive GH findings in this patient, TSH showed negative results. Previous studies (12, 13) have reported that the immunohistochemical staining of TSH in pituitary TSH tumors showed a negative result, but after proteinase K treatment, the immunohistochemical staining of TSH showed positive findings again, which may be insensitive to the antibody used to detect TSH or combined with other factors. In this case, LH was positive and PRL was partially positive in immunohistochemistry, but LH and PRL were not high in hormone detection tests, and there were no corresponding typical clinical manifestations. Thus, the presence of mingling of normal pituitary tissue can be considered during the surgical resection of the adenoma (14). In this case, both pituitary specific transcription factor 1 (PIT-1) and steroidogenic factor-1 (SF-1) were positive and had the ability to secrete growth hormone and thyrotropin. PIT-1 is an important tissue-specific transcription factor that plays a role in determining cell differentiation and hormone production in pituitary adenomas, in regulating the development of anterior pituitary embryo, and in the expression of growth hormone (GH), prolactin (PRL), and thyrotropin (TSH) genes (15, 16). We believe that this co-secretion phenomenon can be explained by the expression of common transcription factors. PIT-1-positive adenomas are also considered biologically aggressive and show a higher risk of recurrence, suggesting that patients should be carefully followed up. Therefore, the diagnosis of mixed pituitary TSH/GH adenoma must be combined with clinical manifestations, immunohistochemical staining and relevant hormone levels, and genetic testing if necessary for comprehensive judgment.

In this case, the patient’s complete examination and whole exome sequencing of the family showed no basis for the genetic syndrome (such as MEN1), suggesting that the incidence of mixed pituitary TSH/GH adenomas may be mostly sporadic. As mixed pituitary TSH/GH adenoma is a rare disease, and the relevant gene detection data are limited, we suggest that if the patient has a family history of pituitary tumor, or the age is less than 30 years, and if the economic conditions allow, clinical lineage whole exome sequencing can be suggested to fill up the gap on the genetic data of mixed pituitary TSH/GH adenomas.

Treatment of mixed pituitary TSH/GH adenomas includes surgery, drug therapy, and radiation therapy, of which the first-line treatment is still surgical resection. Whether pituitary TSHomas or GHomas, most of them are macroadenoma or giant adenoma at the time of diagnosis, often with invasive growth, and the TSHoma tissues usually have high hardness and severe fibrosis, so it brings great challenges to the operation and postoperative remission rate (1). Somatostatin and dopamine receptors can be expressed in both TSHomas and GHomas, especially in mixed pituitary TSH/GH adenoma cells (17). Therefore, the somatostatin analog has become an important drug therapy for the disease. Somatostatin analogues can restore normal thyroid function in about 95% of patients with TSHomas and reduce tumor volume in 50% of patients (8). Luis et al. (18) have found that using somatostatin analogs before surgery not only significantly increases surgical efficacy but also has a high-cost performance and incremental cost-to-effect ratio (ICER). The American Endocrine Society’s clinical guidelines for acromegaly recommend that preoperative somatostatin analogs reduce the risk of surgery in patients with severe pharyngeal thickening, obstructive sleep apnea syndrome (OSAS), or heart failure (19). Therefore, for patients with mixed pituitary TSH/GH adenomas, once the diagnosis is confirmed, the best treatment strategy should be discussed and decided by a multidisciplinary team of experts from endocrinology, neurosurgery, radiotherapy, and neuroimaging. If there are contraindications to surgery or the likelihood of a low success rate, preoperative somatostatin analogs may be considered to control TH and GH levels and prevent complications from improving the postoperative remission rates. If postoperative remission is not complete, radiation therapy or drug therapy is still required (7). In this case, the patient with a mixed pituitary TSH/GH adenoma showed invasive growth and more comorbidities due to the presence of a macroadenoma. It was recommended to use somatostatin analogs to control the size and the hormone levels secreted by the tumor prior to surgery. After using long-acting somatostatin analogs, the hormone levels were well managed, and the comorbidities improved significantly. However, even after 6 months, there was no significant reduction in the tumor size in the pituitary MRI. Therefore, he underwent total pituitary adenoma resection. The postoperative review of the relevant hormone indicators was well recovered. Nevertheless, long-term follow-up is still needed. If the hormone levels cannot be alleviated entirely after the surgery (i.e., the levels of GH, TH, and TSH that are oversecreted before the surgery), and if there is no reduction or the disappearance of tumor on imaging, then radiotherapy or drug treatment is required (6, 7).

In summary, the mixed pituitary TSH/GH adenomas have an insidious onset and slow progression and can present concurrently with clinical features of hyperthyroidism and acromegaly. They often must be distinguished from RTH and, if necessary, require genetic testing. For patients with large adenomas or comorbidities, it is recommended to use somatostatin analogs to restore TH levels and control the excessive secretion of GH levels before surgery. However, the risk of resection of such pituitary adenomas remains high, the rate of complete remission is also low, it is often necessary to combine radiotherapy or drug therapy post-surgery, and lifelong follow-up is recommended.

## Author contributions

LL, LM, YL, RT and XY researched data and/or wrote the manuscript. XL, JZ, JX, ZH and YQ contributed to discussion. All authors contributed to the article and approved the submitted version.
